# Nanoplastics can change the secondary structure of proteins

**DOI:** 10.1038/s41598-019-52495-w

**Published:** 2019-11-05

**Authors:** Oldamur Hollóczki, Sascha Gehrke

**Affiliations:** Mulliken Center for Theoretical Chemistry, University of Bonn, Beringstraße 4, 53115 Bonn, Germany

**Keywords:** Environmental chemistry, Molecular dynamics

## Abstract

Submillimetre-sized plastic particles (microplastics and nanoplastics) of waste origin in the environment have been repeatedly suggested in recent years to have severe impact on living organisms. While the uptake of these materials has been unequivocally evidenced for animals, so far no adverse effects have been observed in the corresponding animal experiments. In this study, we show that nanoplastics are prone to interact with proteins, and this interaction fundamentally changes the functionally crucial secondary structure of these biomolecules, and thereby denaturates them. These results show, for the first time, that the interplay between plastic waste and biological matter can induce significant cellular and thereby ecological damages. Observing these remarkable microscopic level changes highlights the urgent need to extend investigating the effects of these materials through further modelling and molecular biological methods.

## Introduction

The accumulation of plastic waste in the environment has been observed for more than four decades^1^. In the early 2000s, abundant submillimetre-sized fragmentation products (microplastics) of many kinds of plastics were found in the oceans^2^, alarming both environmental scientists and the public. In the following wave of research^3–11^ understanding the environmental consequences of microplastics was aimed at, but the corresponding – and critically discussed^10^ – studies, based almost exclusively on animal experiments, could not identify any direct effects of these materials on living organisms. In accordance, it was argued recently that the more realistic environmental risk arises from even smaller particles with sizes below 100 nm, generally called nanoplastics^4,10^. These particles are at least two orders of magnitude smaller than eukaryote cells, and therefore they can potentially alter living matter on the subcellular or molecular level. While these microscopic effects have not yet been observed directly, the accumulation of polystyrene nanoplastics in a mollusc species has been reported^11^, which infers that these particles can enter the food chain and can be distributed to other marine species, and through sea food eventually to humans as well. Even more importantly, the experiments showed indications that the nanoplastics have diffused through membranes, and have entered even the circulatory system of these organisms^11^.

These shocking findings also indicate that nanoplastics may enter cells. Thus, it is necessary to uncover the interactions of nanoplastics with those biomolecules that occur within cells, since such knowledge will aid us assessing the extent of the structural and functional damage these waste materials can cause in living organisms and in the environment. Recently we have shown that nanoplastics may alter biologically relevant features of lipid bilayers, and thus cell membranes^12^. Among biomolecules, proteins are of particular importance due to the high number of roles they fulfil within living organisms. The sophisticated functions of any given protein are unambiguously defined by its characteristic three-dimensional structure. Changes in the structure can cause defects in these functions, which in some cases can result in the death of the cell and the organism. Thus, when investigating the effects of nanometre-sized materials on biological matter, the protein-nanoparticle interactions must be handled with priority. In the present paper we reveal molecular level effects of nanoplastics on living matter, and demonstrate that the interference of these waste materials with the self-organization of biomolecules through template effects can alter the secondary structure of proteins.

## Results and Discussion

We investigated the interactions of four kinds of plastic with proteins, polyethylene (PE), polypropylene (PP), polyethylene terephthalate (PET), and nylon-6,6 (N66), all abundantly present in nature as both micro- and nanoplastics^2^. The size of the plastic nanoparticles (PNPs) was chosen to be ca. 5 nm, which is expectedly the lower end of the size range that is available in the environment. It is important to stress here that plastics in the environment are unlikely to occur in their pristine form, they should be stabilized from aggregation by molecules adsorbed on their surfaces that are available during their generation. Thus, the mechanism and location of the formation of PNPs is a crucial question. One of their documented origins is the fragmentation within the digestive organs of marine animals^3^. It was recently shown that various microplastic particles with a diameter of ca. 31.5 μm were ground to <1 μm pieces by Antarctic krill^3^. In case of such fragmentation process, assuming spherical particle shapes, 97% of the surface of the new, smaller particles was created within these animals, which means that their surface should get into contact with the molecules available within these organs of the related creatures. Thus, it is reasonable to assume that pristine PNPs are available for and thereafter stabilized by biomolecules, mostly lipids^12^, proteins, protein fragments, and amino acids. Accordingly, the present findings will bear relevance for both the stabilization of nanoplastics, and for their molecular level biological effects.

**Figure 1:**
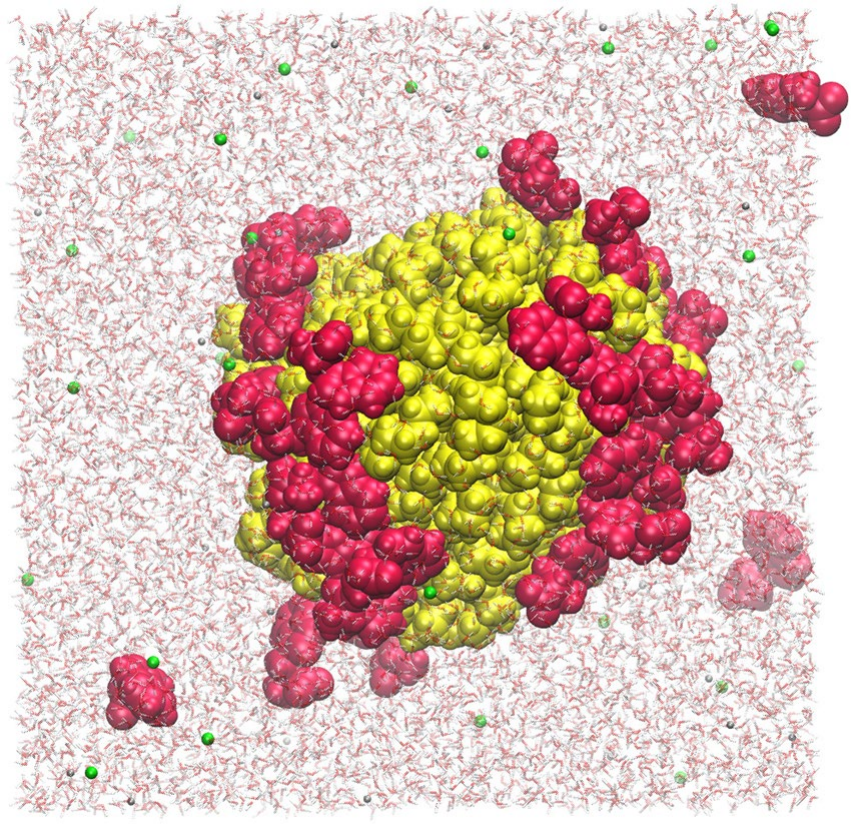
Adsorption of tryptophan (red spheres) on polyethylene nanoplastic (yellow) in water (sticks) in the presence of NaCl (grey and green spheres, respecitvely) and after 20 ns of molecular dynamics simulations. It is visible that the tryptophan molecules adsorb on the surface of the plastic, changing the overall hydrophobicity of the surface of the particle.

To assess if the protein-nanoplastic interactions have any relevance, first the interplay of these nanoparticles with an array of amino acids was tested (glycine, aspartate, arginine, asparagine, phenylalanine, tryptophan). Apparently, the amino acids with non-polar side chains, such as phenylalanine and tryptophan, are prone to adsorb onto the surface of the PNPs. This interaction is so strong, that the PNPs collect nearly all amino acids of this kind from the solutions. In these supramolecular assemblies the hydrophobic side chain points to the surface of the similarly hydrophobic PNP, whilst the amino and carboxylate groups of the amino acids point toward the aqueous solution (Fig. 1). Forming such a micelle-like structure around the PNPs shows that the hydrophobic nature of nanoplastics can be masked by biomolecules, which will affect their solubility, and their aggregation behaviour. Even more importantly, these results reveal that the association of plastic particles and biomolecules are directed based on the same mismatch of hydrophobicity and hydrophilicity as the supramolecular self-organisation of biomolecular systems (e.g. membranes, and proteins); therefore, these nanoparticles are likely to directly incorporate into, and thereby interfere with the functionally crucial molecular level structure of living matter.

According to the findings above, it is reasonable to assume that the strong PNP-amino acid interplay may interfere with the folding of proteins. To test this hypothesis, we performed molecular dynamics simulations on two peptides, which represent the two most important kinds of secondary structures in proteins. The first of these was a tryptophan zipper^13^, which has a β-hairpin structure, and resembles β-sheets in proteins. Such β-hairpins were suggested to form nucleation sites for protein folding, which makes investigating the effects of PNPs on them highly important. The second peptide was an α-helix polypeptide of 12 alanine amino acids. Alanine is intrinsically stabilising α-helices^14–16^, therefore this species is highly stable, and any changes occurring in its secondary structure would indicate the general sensitivity of helices toward nanoplastics.

**Figure 2:**
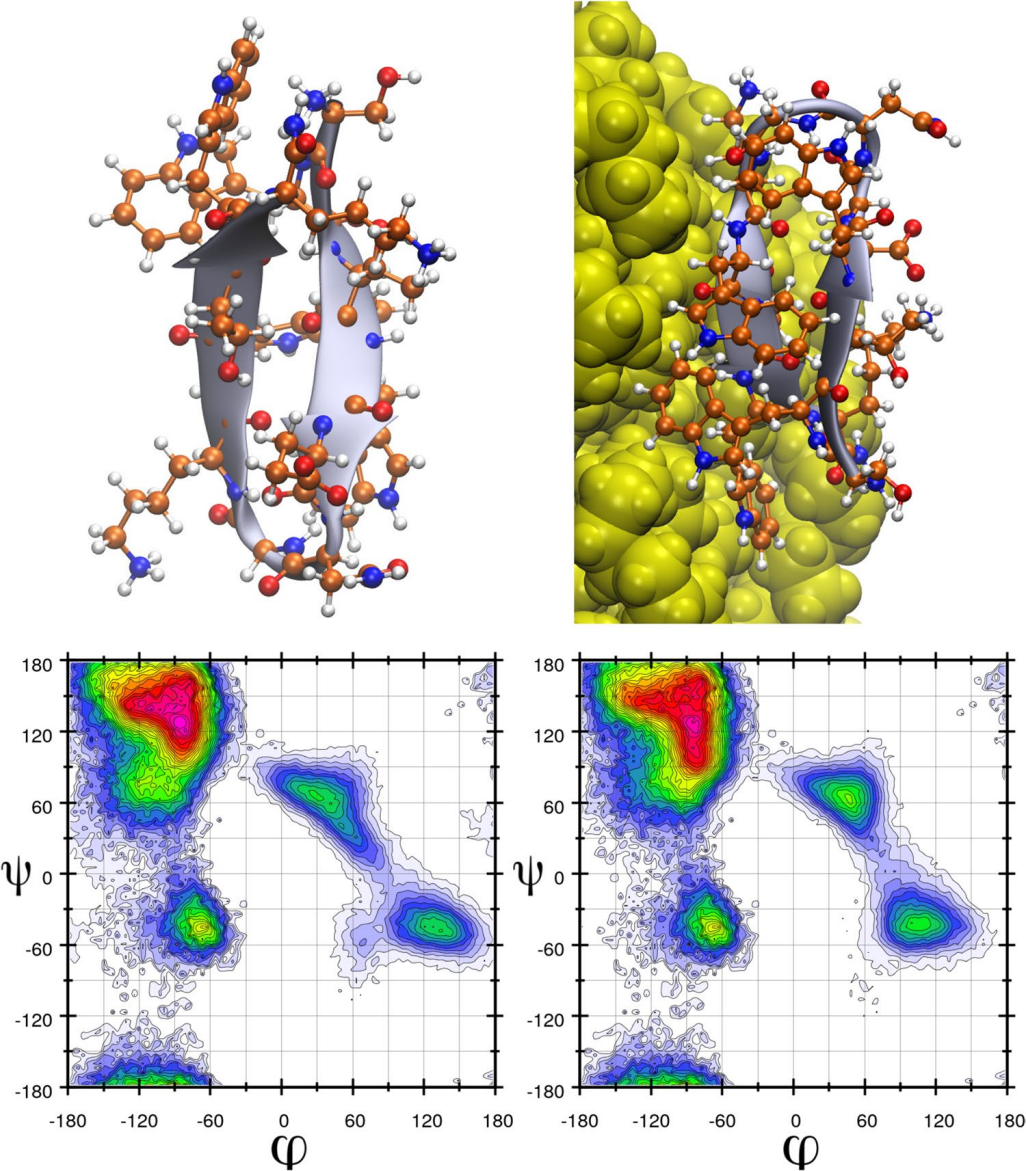
Three dimensional structure (above) and Ramachandran plot (below, populations are indicated through a colour code decreasing as red > blue > white, for scales, see Suppl. Info.) of tryptophan zipper in the absence (left) and presence (right) of a polyethylene nanoplastic particle. It is visible that although the peptide is adsorbed on the surface of the plastic, its overall structure is not altered by the interaction.

**Figure 3:**
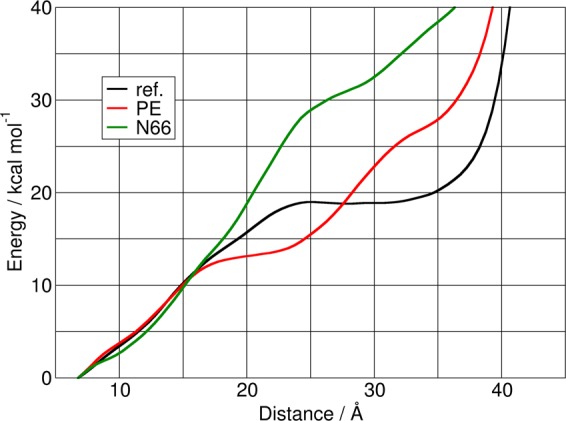
Relative free energy of the tryptophan zipper versus the distance between the C-terminal carboxyl carbon atom and the N-terminal nitrogen atom, obtained as described in the methodology section. The curves are produced in the absence of nanoplastic particles (black) as reference, and in the presence of a polyethylene (red) or a nylon-6,6 particle (green). The differences between the curves clearly indicate the effect of plastics on the conformational changes of the peptide.

The tryptophan zipper adsorbs on the surface of all investigated PNPs, which is in good agreement with the aforementioned strong interplay between tryptophan and the plastics. This interaction does not induce any significant spontaneous changes in the peptide structure, as compared to that in the absence of the plastics (Fig. 2). The lack of structural reorganisation, however, does not necessarily mean that the plastic has no influence on the secondary structure of the peptide, and it is conceivable that the rearrangement is kinetically hindered, and therefore too slow to observe in the time scales available for molecular dynamics simulations. Such slow processes can be investigated through potential of mean force calculations (for details, see the section Methods and Models), which provide information on the energetics of the system as a function of a well-defined coordinate. For this β-hairpin structure, the most characteristic structural feature is the folding back of the protein backbone into a loop-like shape. This shape requires the C- and N-terminal of the peptide to be closer to each other than in a hypothetical α-helical isomer. For this reason, we chose to characterize the stability of this β-hairpin structure through the energetics of increasing the distance between the C-terminal carboxyl carbon atom and the N-terminal nitrogen atom (labelled here as d_C-N_). The free energy demand of increasing this distance shows interesting trends. In the absence of the PNPs the free energy constantly increases until ca. d_C-N_ = 25 Å, where it levels out at 19 kcal mol^−1^, which is the energy demand of breaking all the intramolecular hydrogen bonds within the peptide (Fig. 3). After ca. d_C-N_ = 35 Å, the free energy exhibits a steep increase, which can be attributed to the stretching of the covalent bonds of the peptide backbone after reaching a completely linear conformation (Fig. 3).

Until ca. d_C-N_ = 15 Å the presence of the PNPs has no significant effect on the detachment of the two terminal atoms. PE apparently stabilizes a conformation at d_C-N_ = 20 Å at 13 kcal mol^−1^, which is ca. 4–5 kcal mol^−1^ lower in energy than the corresponding structure in the absence of the plastic. After further stretching, however, the free energy rises more steeply, and at d_C-N_ = 27.6 Å it becomes destabilized compared to the reference system. These results can be rationalized through the facilitated partial opening of the hairpin in the presence of PE, and the destabilization of the fully open structure. Interestingly, in the presence of N66 a completely different behaviour can be observed. With growing distance between the two terminal atoms beyond d_C-N_ = 15 Å, the free energy of the system increases monotonously, and compared to the reference system the fully linear structure (at d_C-N_ = 31 Å) is by 13 kcal mol^−1^ destabilized (Fig. 3). In other words, the presence of the nylon nanoparticle apparently hinders the disintegration of the β-hairpin structure.

**Figure 4:**
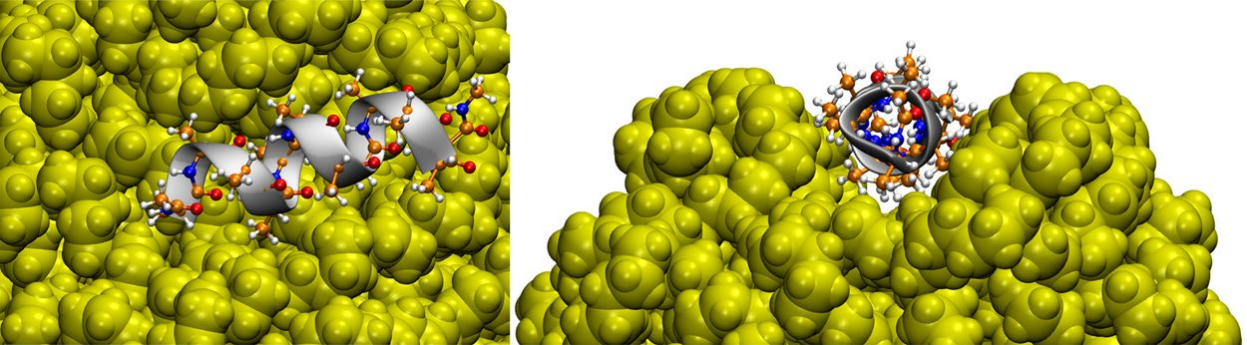
Three dimensional structure of the helical peptide (composed of 12 alanine units) on the surface of a polyethylene nanoparticle (yellow) from two views, at the end of the 20 ns simulation at 353 K. It is visible how the plastic rearranges to encompass the peptide.

The α-helix composed of 12 alanine units is also found to adsorb on the predominantly hydrophobic surface of the PNPs. Helical protein moieties are often adjacent to hydrophobic species in biological systems, such as the transmembrane domains of membrane proteins, in agreement with these findings. In case of the most hydrophobic, PE and PP PNPs, the somewhat mobile polymer chains at the surface of the PNP rearrange in a manner that a pocket is formed, which thereby allows encompassing more of the peptide. Very likely, the affinity of the polymer chains to the peptide in longer timescales allows an even larger rearrangement of the plastic nanoparticle, which would then fully surround the α-helix, isolating it from the solution. To verify this hypothesis, we performed another simulation with the helical peptide adsorbed on the surface of the polyethylene nanoparticle at 353 K. At this higher temperature, the higher kinetic energy of the atoms allows for a faster movement and a faster rearrangement of the otherwise sluggish molecules. Interestingly, while the secondary structure of the protein was retained, the polymer chains moved more toward encompassing the helix (Fig. 4). If the plastic indeed shows propensity to surround such peptide structures, it may penetrate the space in between α-helices of a protein, and thereby it may induce severe changes in the tertiary structure of these biomolecules.

**Figure 5:**
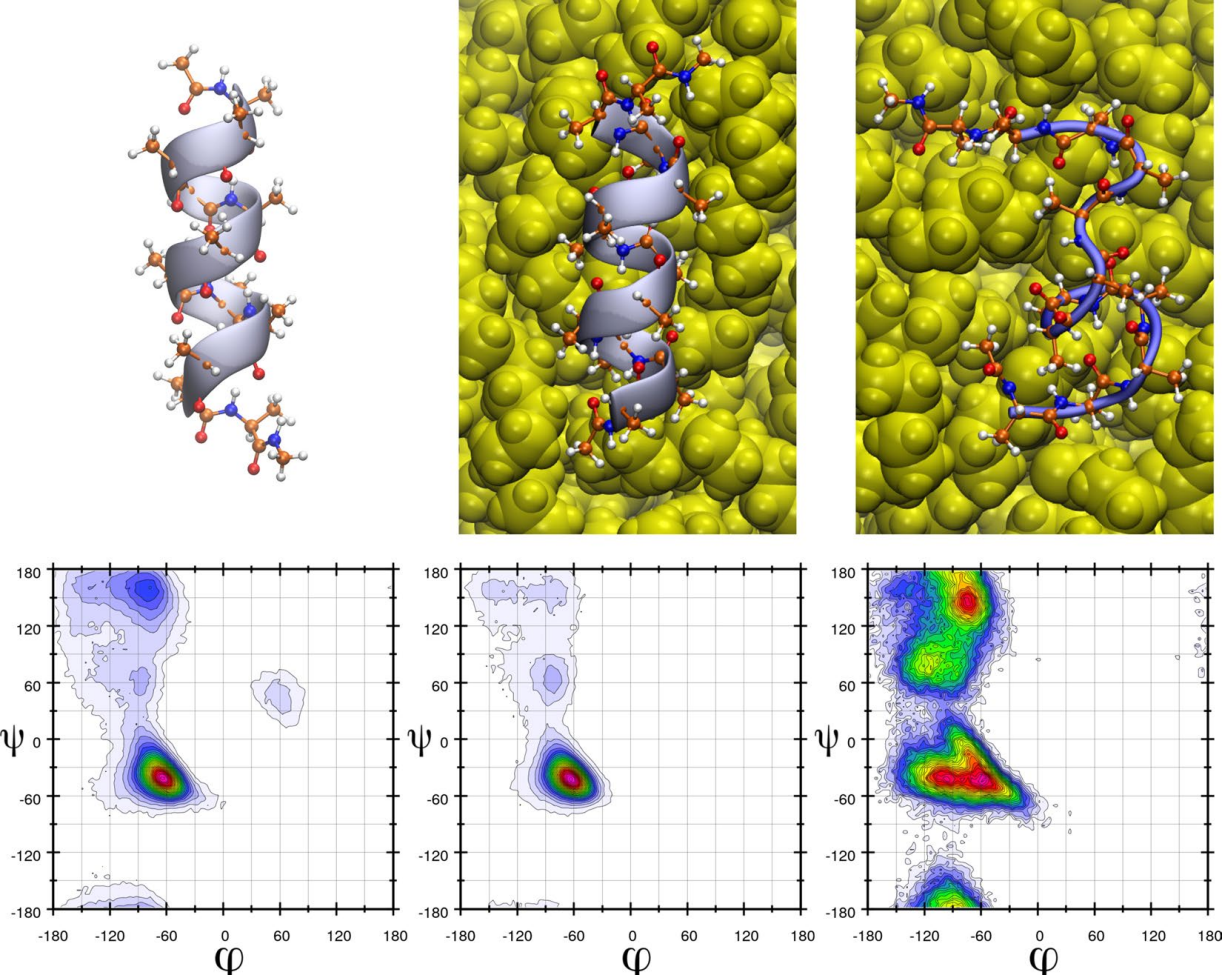
Three dimensional structures (above) and Ramachandran plots (below, populations are indicated through a colour code decreasing as red > blue > white, for scales, see Suppl. Info.) of the α-helical peptide composed of 12 alanine amino acids in the absence of plastics (left), and in the presence of a polyethylene (middle) and nylon-6,6 (right) nanoplastic particle. It is visible that the α-helical structure is enhanced in the presence of polyethylene as the β-sheet-like areas in the Ramachandran plot become less populated, while the nylon unfolds the helix, and – based on the Ramachandran plots – changes it into a more β-sheet-like structure.

In most cases the overall structure of the helix barely changes upon adsorption, and in fact, by hindering the mobility of the two terminal amino acids, the helical structure is even stabilized further (see Ramachandran plots on Fig. 5). On the nylon particle, however, severe changes in the α-helix can be observed (Fig. 5). Under closer scrutiny, the helical backbone of the peptide appears to have changed spontaneously into a β-loop-like structure, as proven by the Ramachandran plots. Due to the aforementioned intrinsically high stability of polyalanine α-helices, it is reasonable to assume that other helices would also undergo a similar transformation. The most conspicuous feature of this plastic that distinguishes it from the others is the presence of the amide moieties on its surface. These groups offer a set of hydrogen bond donor and acceptor sites at the surface to proteins, which are expectedly similar in strength to the intramolecular protein-protein hydrogen bonds. The possibility of forming such peptide-plastic interactions results in a strong competition to those hydrogen bonds, which are necessary for the integrity of the helical structure, and thereby define the secondary structure of the peptide. Since the amide groups of the plastic are separated by hydrophobic units, interaction sites on the surface of the PNP are scattered (see Suppl. Inf.), so the peptide must change its conformation to maximize its interactions with the surface. In other words, the plastic forms a template for the peptide, to which it is forced to adjust its own structure. This template effect should be, of course, dependent on the location on the surface, since the position of the amide moieties relative to each other on the otherwise non-polar surface varies (see Supp. Inf. Fig. S6). While – as seen above – some points at the surface should offer a template that is highly prone to induce the rearrangement of the secondary structure of peptides, some areas could preserve it relatively intact. In agreement, in a simulation from a different starting geometry we observed another adsorption mode, which retained the native structure of the peptide.

Thus, nanoplastics affect the folding of proteins. In case of interacting with fully hydrophobic plastics, such as polyethylene, the α-helical structures appear to be favoured, as the partial cleavage of the β-hairpin structure of a tryptophane zipper was found to be thermodynamically less demanding, while polymer chains on the plastic surface were found to reorient in order to harbour the α-helical structures more intensively. In the presence of nylon, however, the opposite effect was observed, and a fully α-helical structure changed spontaneously into a β-loop-like conformation on the surface of the corresponding plastic nanoparticle. The importance of these findings points beyond the mere destructive effect of plastics on proteins. There are similar conformational changes that are known to induce an organism-wide chain reaction of autocatalytic peptide denaturation. For instance, the prion disease bovine spongiform encephalopathy^17,18^ can be traced back to transforming the membrane protein PrP^C^ into the scrapie PrP^Sc^ through converting α-helix residues into β-sheet structures^17,18^. Similarly, in Alzheimer’s disease the analysis of amyloid filaments indicated an induced β-sheet formation of helical proteins^19^, which was linked to the abnormal plaque formation in neural tissues, further showcasing the gravity of induced changes in the secondary structure of proteins. Since our simulations showed here the same kind of transformation for the model peptides, it is reasonable to assume that the molecular biological effects of nanoplastics are more severe than thought before, urgently requiring further molecular biological, theoretical and ecological studies.

About the same time as our initial submission, Kihara *et al*. and Gopinath *et al*. published experimental studies on nanoplastics-protein interactions^20,21^. They observed the formation of coronae on PNPs through the adsorption of proteins on the plastic surface^20,21^. In this interaction, the secondary structure of proteins was indeed changed, and thereby they were denaturated^21^. This adsorption, in fact, increased the aggregation of the nanoparticles, and triggered an immune response^21^. While these results clearly confirm our theoretical findings, and show the actual macroscopic biological effects, our present simulations show the actual mechanism of these processes on a molecular level, and therefore the two sets of results complete each other.

## Models and Methods

All simulations presented in this study were performed with the LAMMPS program^22^. Water molecules were modelled with the SPC/E water model^23^, while the plastic, amino acid and peptide molecules with the atomistic OPLS-AA force field^24^. The plastic nanoparticles were created through folding single polymer chains of the corresponding plastics by a series of molecular dynamics simulation and energy minimization steps. For polyethylene 16 C_72_H_146_ chains, for the polypropylene 8 C_144_H_290_ chains, for polyethylene terephthalate 8 C_122_H_102_O_50_ chains, and for nylon 8 C_156_H_288_N_26_O_27_ chains were taken. Starting from their fully linear conformation, the chains were aligned next to each other and simulated under periodic boundary conditions at 500 K and under 1 bar pressure in an isothermal-isobaric (NpT) ensemble, using Nosé–Hoover chain thermostat and barostat for 5 ns, in order to generate randomly aligned polymer chains. The last snapshot of this simulation was used in the further steps. Each obtained polymer chain assembly was placed into a larger, cubic simulation box with cell vectors of 100–150 Å, containing only the plastics. These boxes were thereafter simulated in an NVT ensemble at three different temperatures (300 K, 400 K, and 500 K) each for 1.5 ns, after which they were cooled down gradually over a 10 ns molecular dynamics run to 200 K. Finally, an energy minimization was performed on each of the resulting structures, and the total energies were compared. For the subsequent molecular dynamics simulations the structures with the lowest total energy were chosen for each plastic. The selected plastic particles were all close to a spherical shape, with a diameter of ca. 5 nm.

The simulation boxes containing the nanoplastics and the amino acids were created by taking the solubility of the amino acids in water into consideration. The plastic particle, 10000 water molecules, 30 amino acid molecules, and 28 sodium and chloride anions were placed into the simulation box. In case of amino acids bearing charge (arginine and aspartate) extra chloride or sodium ions were given to ensure charge neutrality. The amino acids were included in their zwitterionic form, thus, with a deprotonated carboxyl group, and a protonated amino group, which corresponds to aqueous solutions at neutral pH. The structure of the tryptophan zipper was downloaded from the Protein Data Bank (PDB ID: 1LE1), where it was deposited by Cochran *et al*.^13^. The simulation boxes with the peptide models (tryptophan zipper and polyalanine α-helix) contained a single plastic particle, a single molecule of the peptide, 28 sodium ions, and 28 chloride anions, and 10000 water molecules. For the tryptophan zipper an extra two chloride anions were given to maintain charge neutrality.

The simulation boxes were created by using the Packmol program^25^, with initial cell vectors that correspond to ca. 0.8 g cm^−3^ density. After an initial energy minimization, the systems were simulated for 1 ns in an NpT ensemble at a temperature of 293 K and under 1 bar pressure by using a Nosé–Hoover thermostat and barostat. The volume of the box was averaged for the last 0.5 ns of this simulation, which was used for the volume of the boxes for the subsequent molecular dynamics runs, for which we switched to a canonical (NVT) ensemble with the same settings for the thermostat. After 3 ns of equilibration, a 20 ns long production run was performed. For creating the Ramachandran plots, the TRAVIS program was applied^26^.

The simulations aimed at obtaining the potential of mean force curves of Fig. 3 were conducted similarly to the description above. The terminal atoms of the tryptophan zipper were kept with an external harmonic potential to 4 Å. After the NpT simulations and the 3 ns of equilibration in a canonical ensemble as above, a 0.25 ns production run was performed, during which the forces that affected along the distance of these two atoms were averaged. Thereafter, the forced distance of the terminal atoms was increased gradually by 0.5 Å increments, performing at every distance 0.25 ns of equilibration and 0.25 ns of production run, during which the corresponding forces were again averaged. Integrating these forces provided the potential of mean force curves shown in Fig. 3.

All additional data and details can be found in the Supporting Information.

## Supplementary information

Supplementary Information
