# Supplementary material for: Nanoplastics can change the secondary structure of proteins
Source: Sci Rep. 2019 Nov 5;9:16013. doi: 10.1038/s41598-019-52495-w (PMC6831684; doi:10.1038/s41598-019-52495-w)
Supplement: Supplementary file 1 — Supplementary Information [file 41598_2019_52495_MOESM1_ESM.pdf]

Nanoplastics can change the secondary structure of proteins

Oldamur Hollóczki,* Sascha Gehrke
Mulliken Center for Theoretical Chemistry, University of Bonn, Beringstraße 4, 53115 Bonn, Germany
e-mail: holloczki@thch.uni-bonn.de

Supporting Information

Abbreviations used in this document:
PE: Polyethylene
PP: Polypropylene
PET: Polyethylene terephthalate
N66: Nylon-6,6
Neat: In the absence of plastic nanoparticles

Table of Contents

1. Structural images of the nanoparticles generated and used in the present study............................S2
2. Ramachandran plots of the α-helix and tryptophane zipper structures in aqueous solutions, and adsorbed on the plastic nanoparticles, together with structural images..S4
 Neat α-helix and tryptophan zipper (in the absence of plastics)..S4
 α-Helix on polyethylene...S5
 α-Helix on nylon-6,6..S5
 α-Helix on polypropylene...S6
 α-Helix on PET...S6
 Tryptophan zipper on polyethylene...S7
 Tryptophan zipper on nylon-6,6..S7
 Tryptophan zipper on polypropylene...S8
 Tryptophan zipper on PET..S8
 Comparison of the α-helix structures on the different plastics through rescaled Ramachandran plots, with identical scaling...S9
 Comparison of the tryptophan zipper structures on the different plastics through rescaled Ramachandran plots, with identical scaling...S10
3. Time development of the surface of the nanoplastics covered by the selected amino acids and water in the MD simulations..S11
 Amino acids at polyethylene...S12
 Amino acids at polypropylene...S13
 Amino acids at polyethylene terephthalate..S14
 Amino acids at nylon-6,6..S15

1. Structural images of the nanoparticles generated and used in the present study

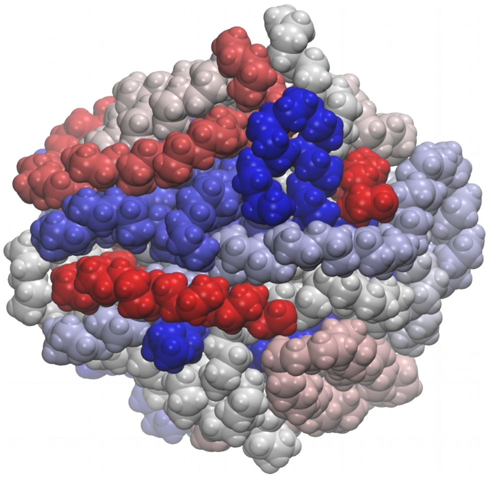

Figure S1: PE Nanoparticle used in the simulations. Each chain is highlighted in a different color.

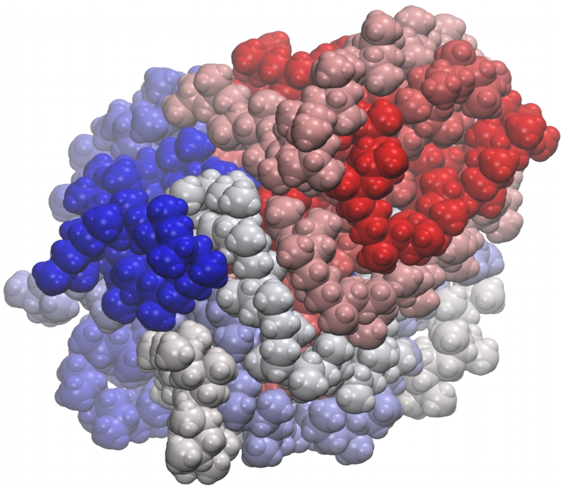

Figure S2: PP Nanoparticle used in the simulations. Each chain is highlighted in a different color.

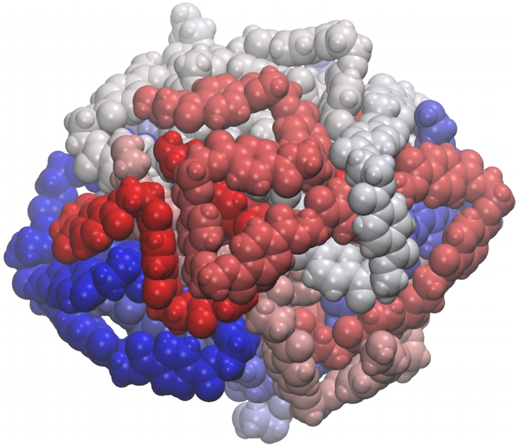

Figure S3: PET Nanoparticle used in the simulations. Each chain is highlighted in a different color.

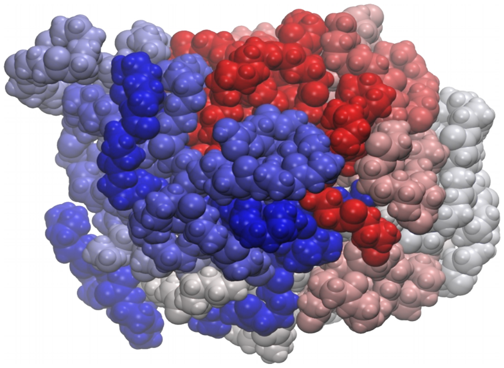

Figure S4: N66 Nanoparticle used in the simulations. Each chain is highlighted in a different color.

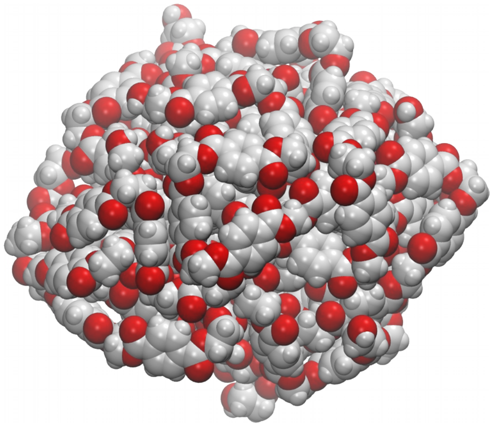

Figure S5: PET Nanoparticle used in the simulations. Oxygen atoms are highlighted in red, showing the scattered interactions sites at the surface of the nanoparticle

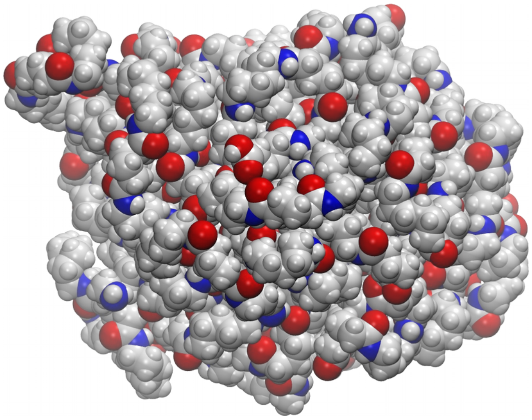

Figure S6: N66 Nanoparticle used in the simulations. The oxygen atoms are highlighted in red, the nitrogen atoms are in blue. The surface shows a distinct scatter of hydrogen bond donor (N-H) and acceptor sites (O).

2. Ramachandran plots of the α-helix and tryptophane zipper structures in aqueous solutions, and adsorbed on the plastic nanoparticles

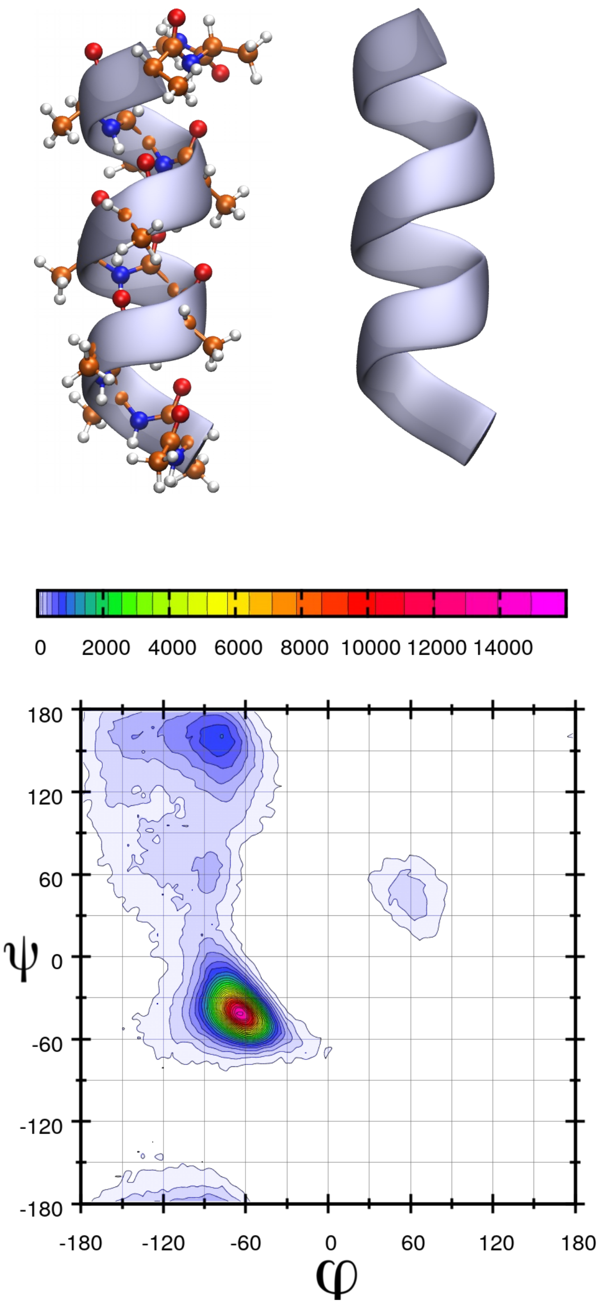

Figure S7: Free α-helix structure in saline solution

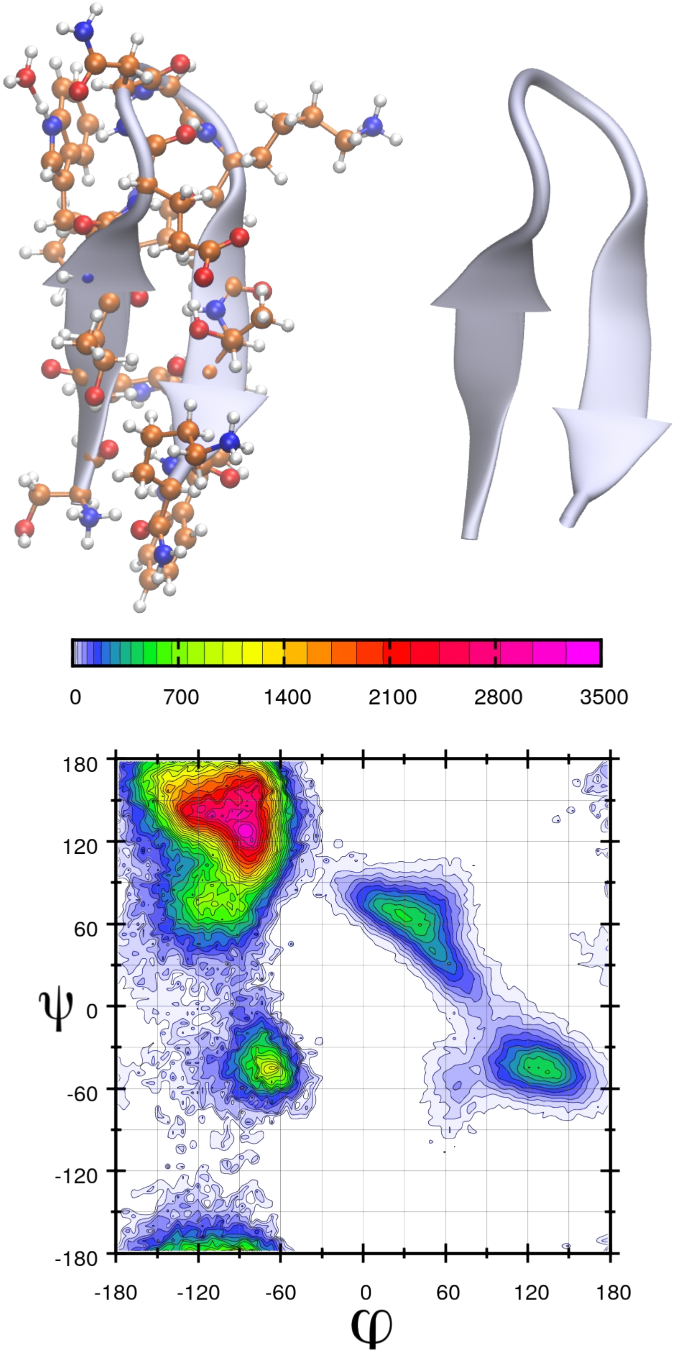

Figure S8: Free tryptophan zipper structure in saline solution

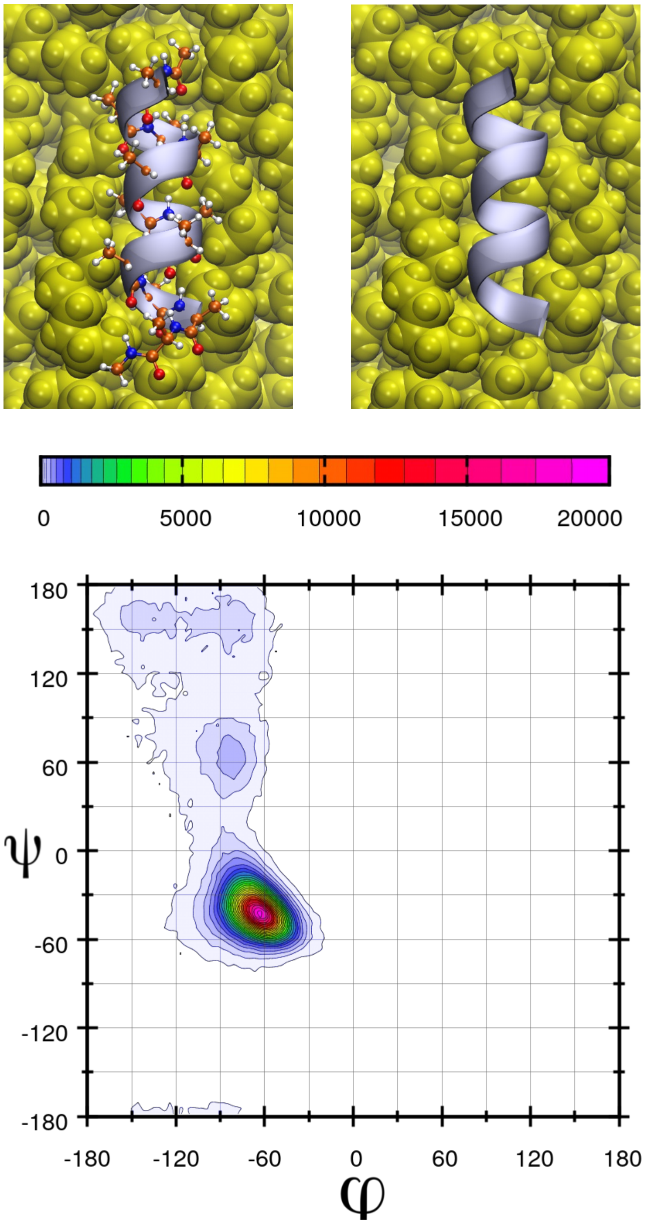

Figure S9: α-Helix structure on PE nanoparticle

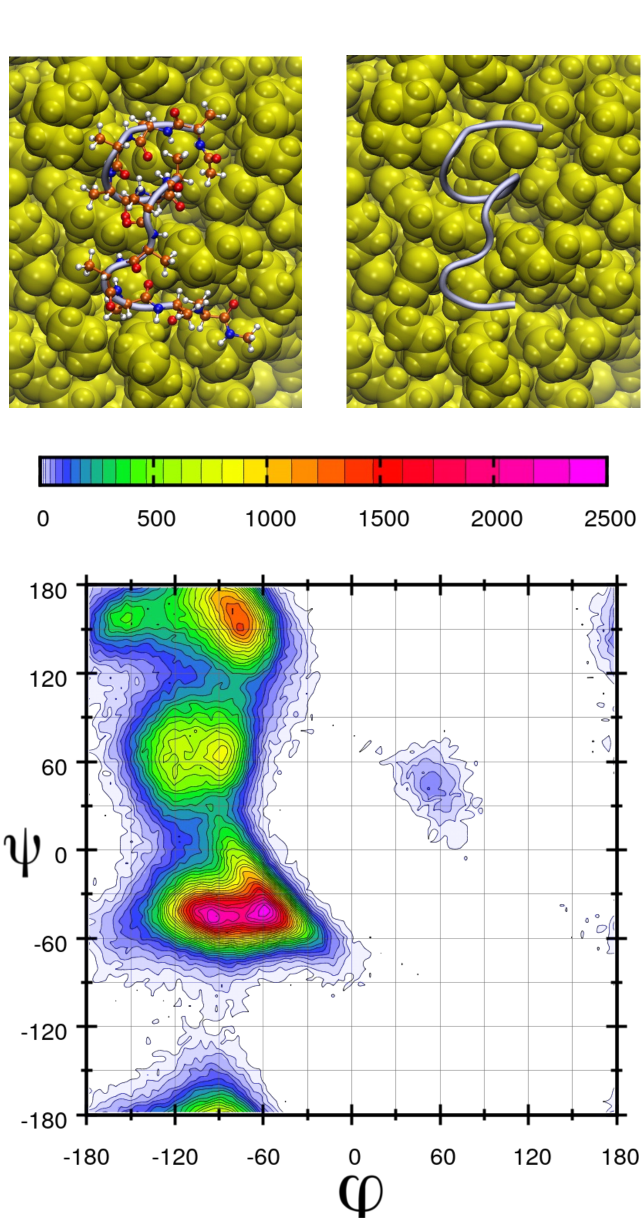

Figure S10: α-Helix structure on N66 nanoparticle

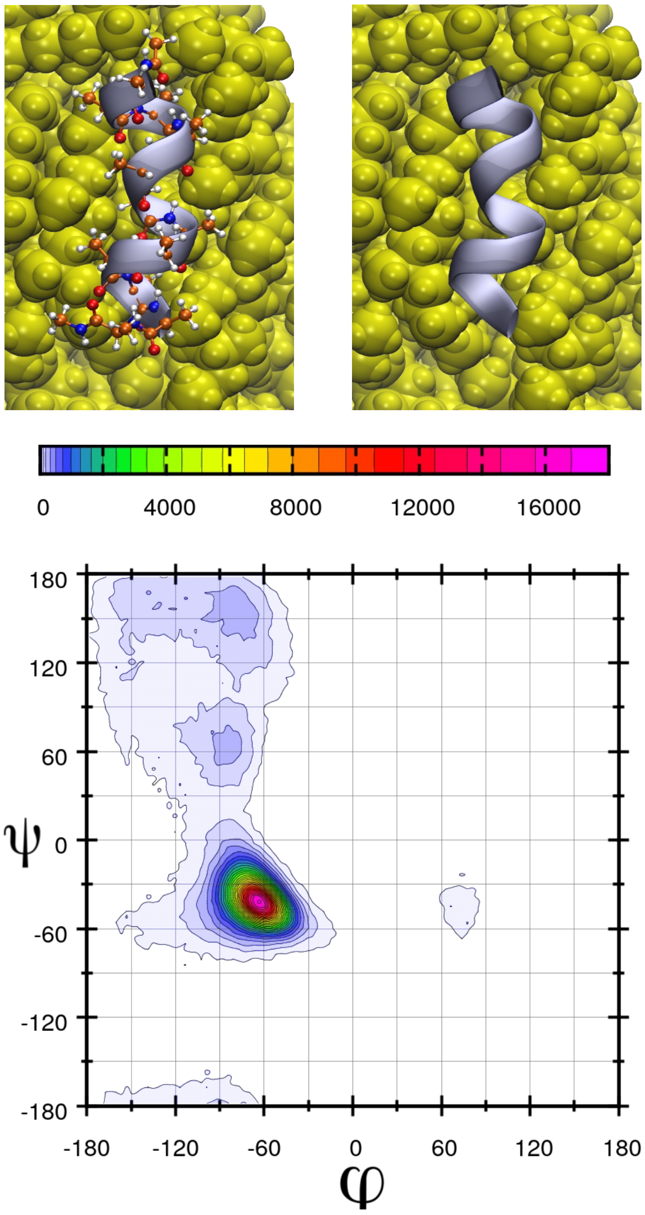

Figure S11: α-Helix structure on PP nanoparticle

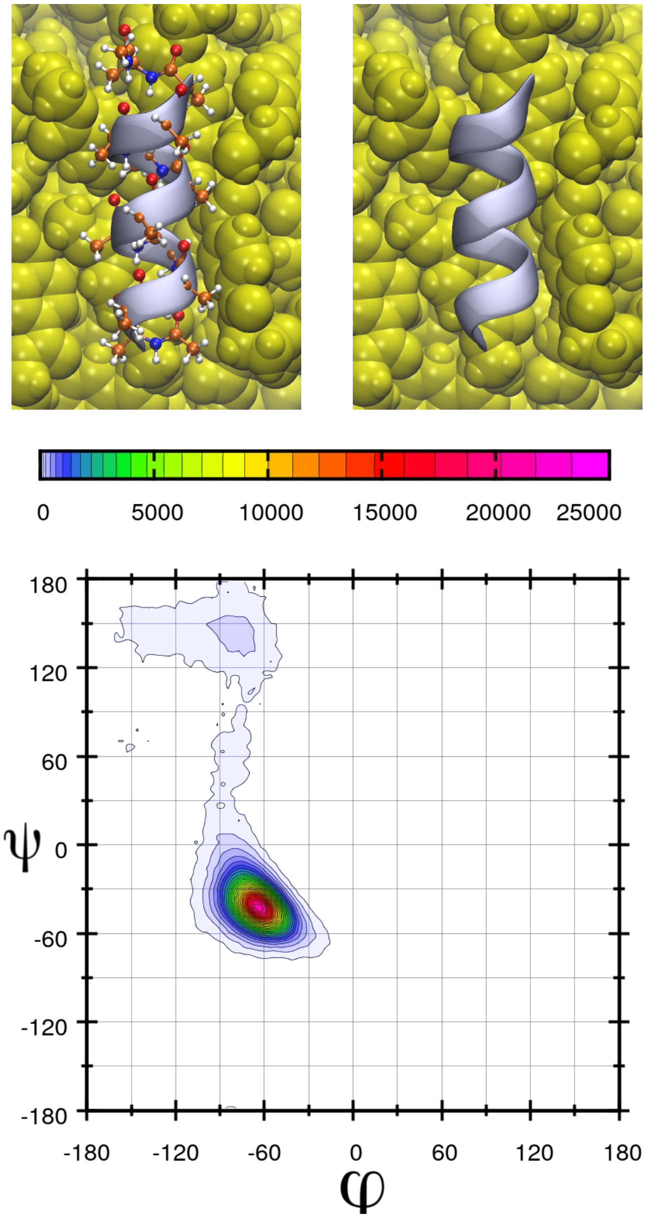

Figure S12: α-Helix structre on PET nanoparticle

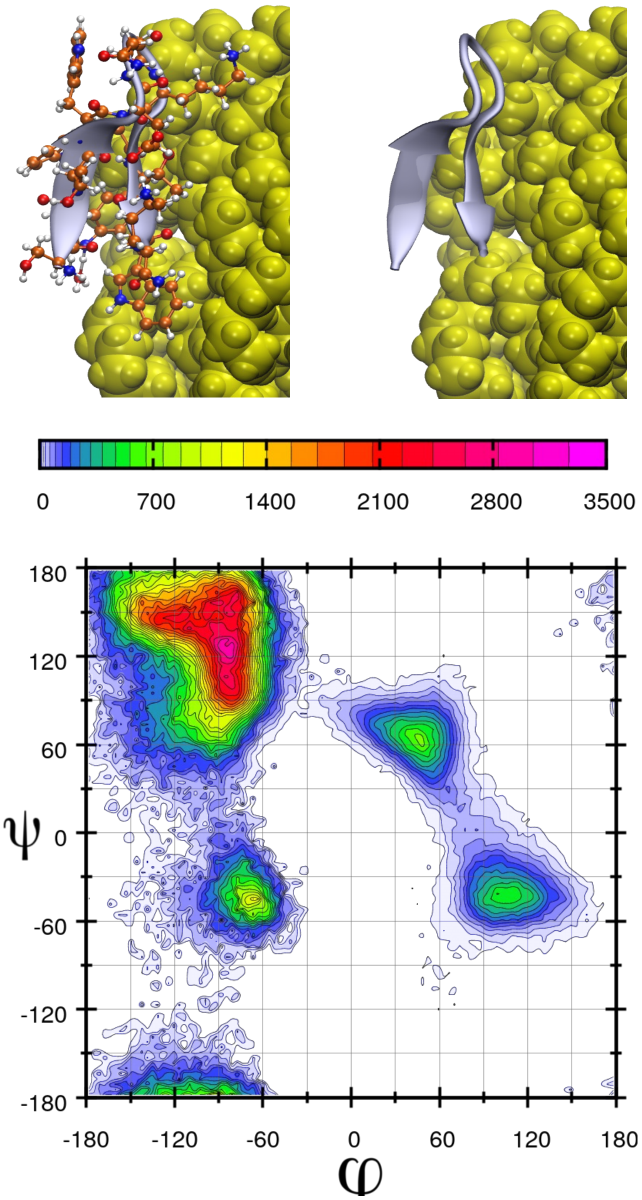

Figure S13: Tryptophan zipper structure on PE nanoparticle

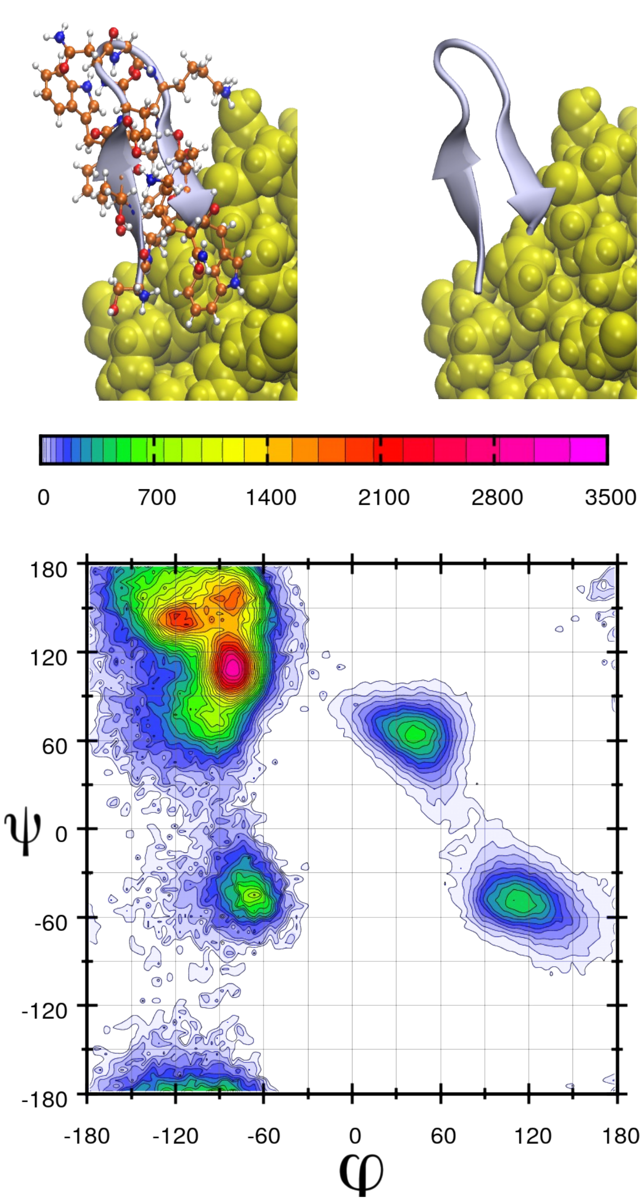

Figure S14: Tryptophan zipper structure on N66 nanoparticle

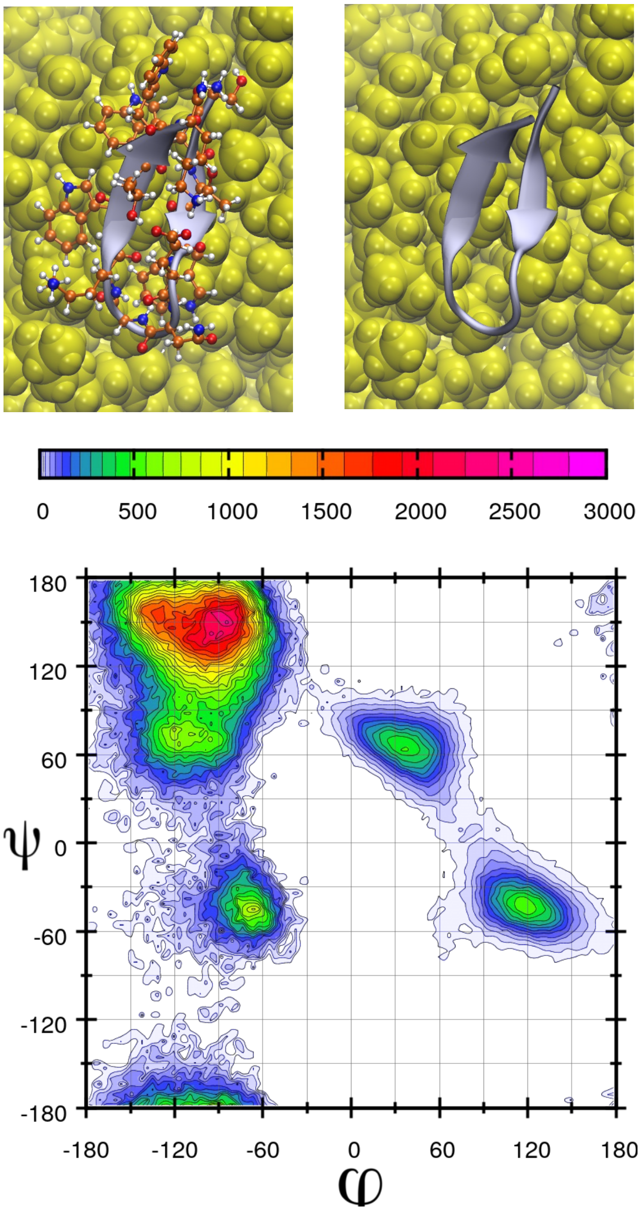

Figure S15: Tryptophan zipper structure on PP nanoparticle

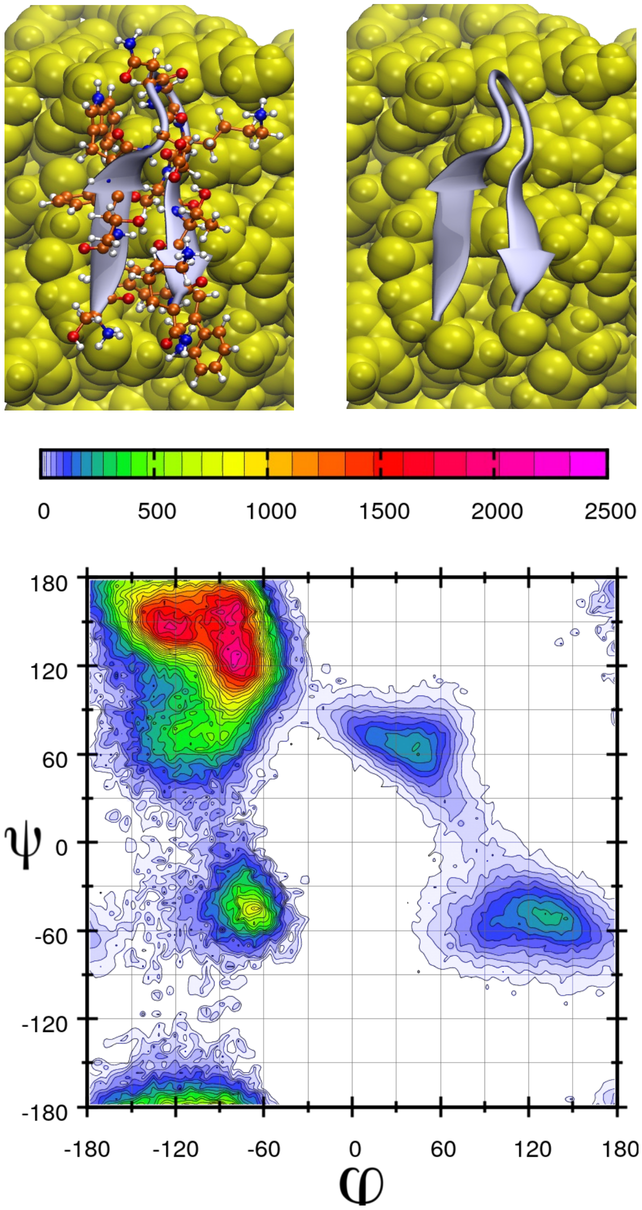

Figure S16: Tryptophan zipper structure on PET nanoparticle

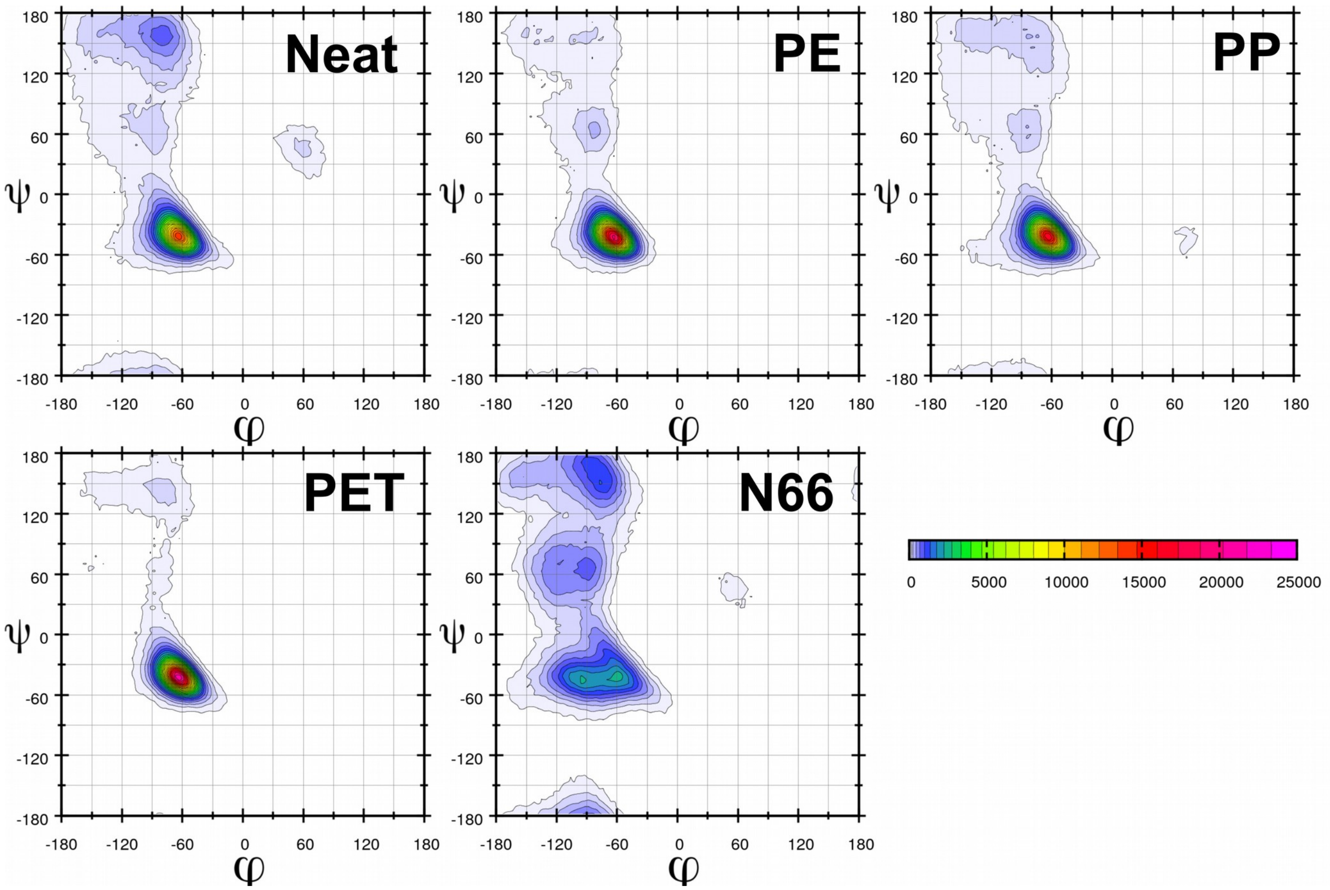

Figure S17. Rescaled Ramachandran plots of the α-helix structures with identical scaling allowing direct quantitative comparison of the plots

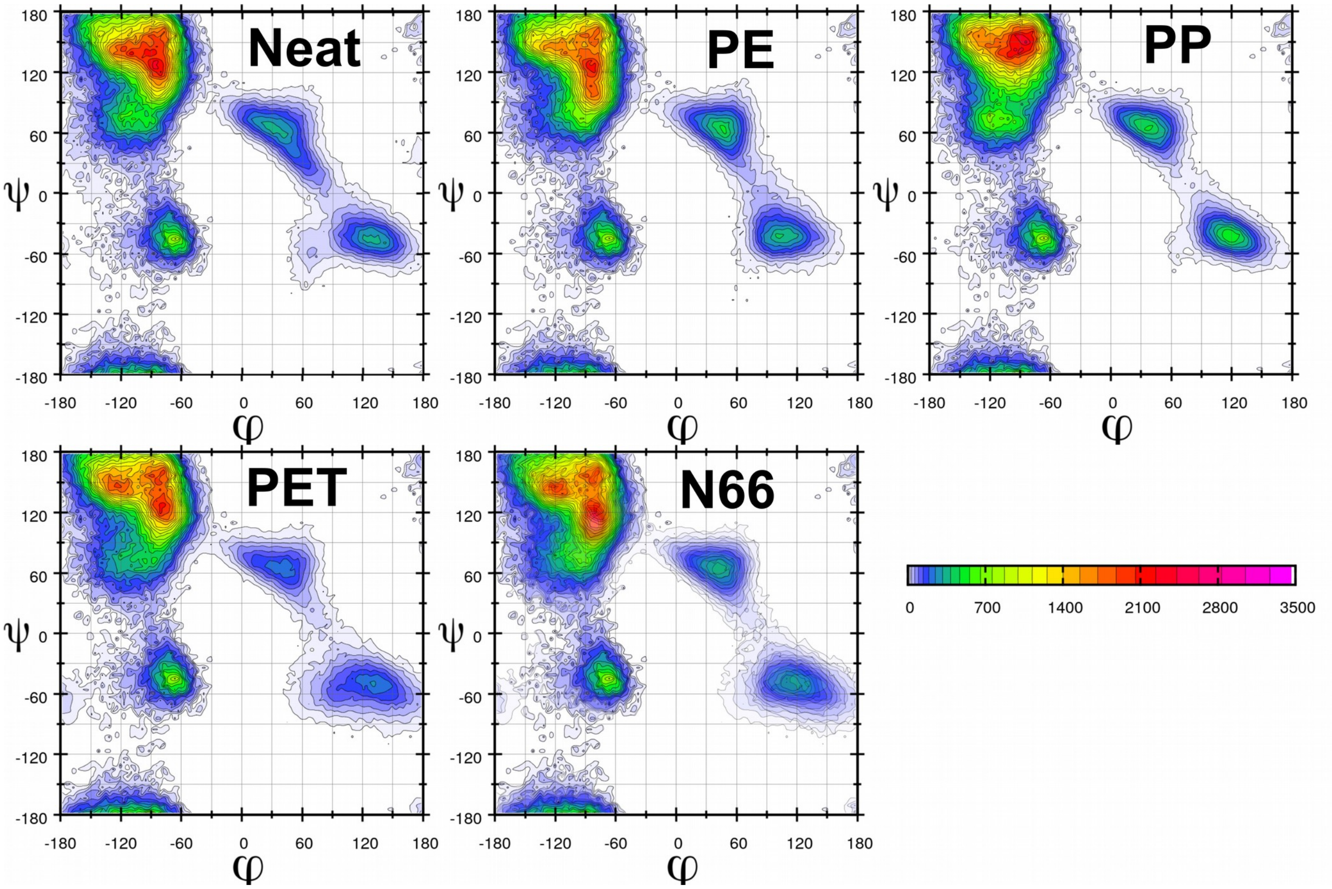

Figure S18. Rescaled Ramachandran plots of the tryptophane zipper structure with identical scaling allowing direct quantitative comparison of the plots

3. Time development of the surface of the nanoplastics covered by the selected amino acids and water in the MD simulations

Simulations of the plastic particles in aqueous saline solutions in the presence of six selected amino acids (glycine, aspartate, arginine, asparagine, phenylalanine, tryptophan) were performed. In one simulation box, one plastic nanoparticle, and one kind of amino acids were present, resulting in altogether 24 simulations. The further details are given in the Methods and Models section of the article.

The graphs shown in this section present the interaction surfaces of the plastic nanoparticle with the surrounding aqueous solution, and with the amino acids for each simulations. These surfaces were calculated through Voronoi tesselation at every picosecond of the simulation, as implemented in the Travis code. High interaction surface of the nanoparticles with the amino acid shows that the amino acid adsorbs on the surface of the plastic, which occurs almost exclusively in case of the amino acids with non-polar (hydrophobic) side chains. It is also possible to observe that the total surface of the plastic particle increases upon the adsorption of these amino acids.

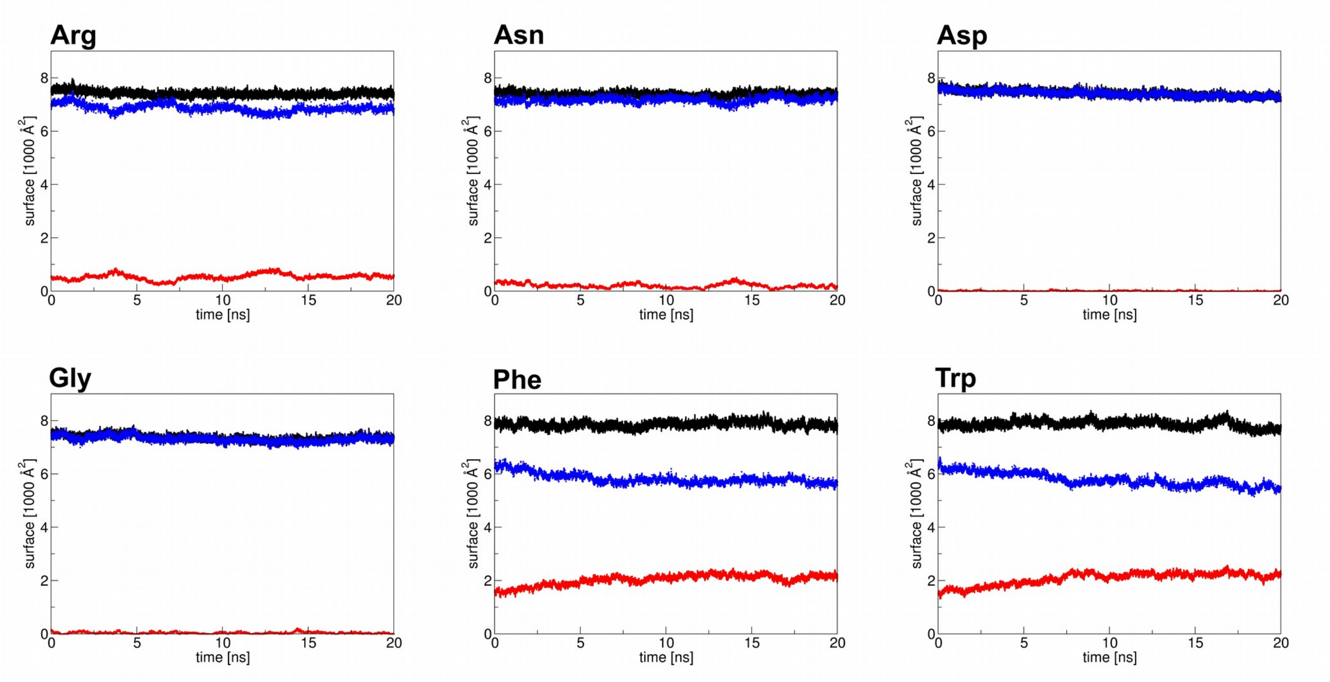

Figure S19. Time development of the total surface area (black), and surface coverages of the ***polyethylene*** *nanoparticle by water (blue) and the amino acids (red) in the molecular dynamics simulation.*

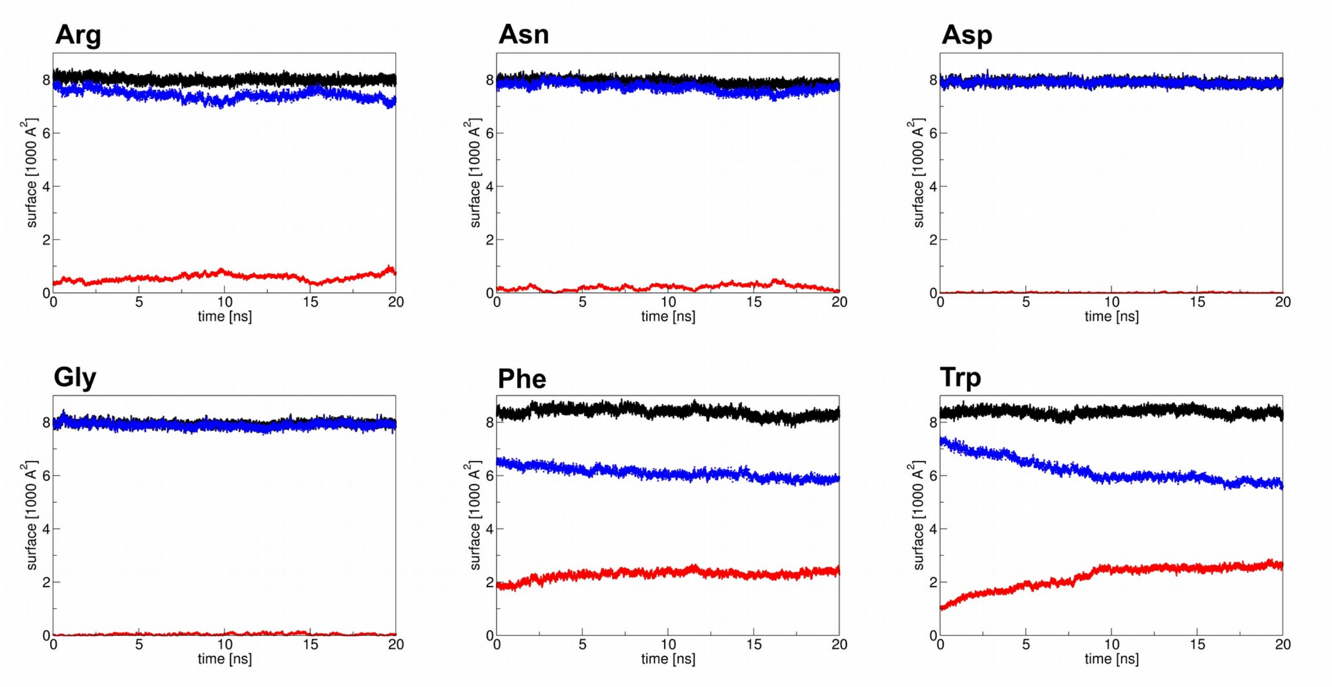

Figure S20. Time development of the total surface area (black), and surface coverages of the ***polypropylene*** *nanoparticle by water (blue) and the amino acids (red) in the molecular dynamics simulation.*

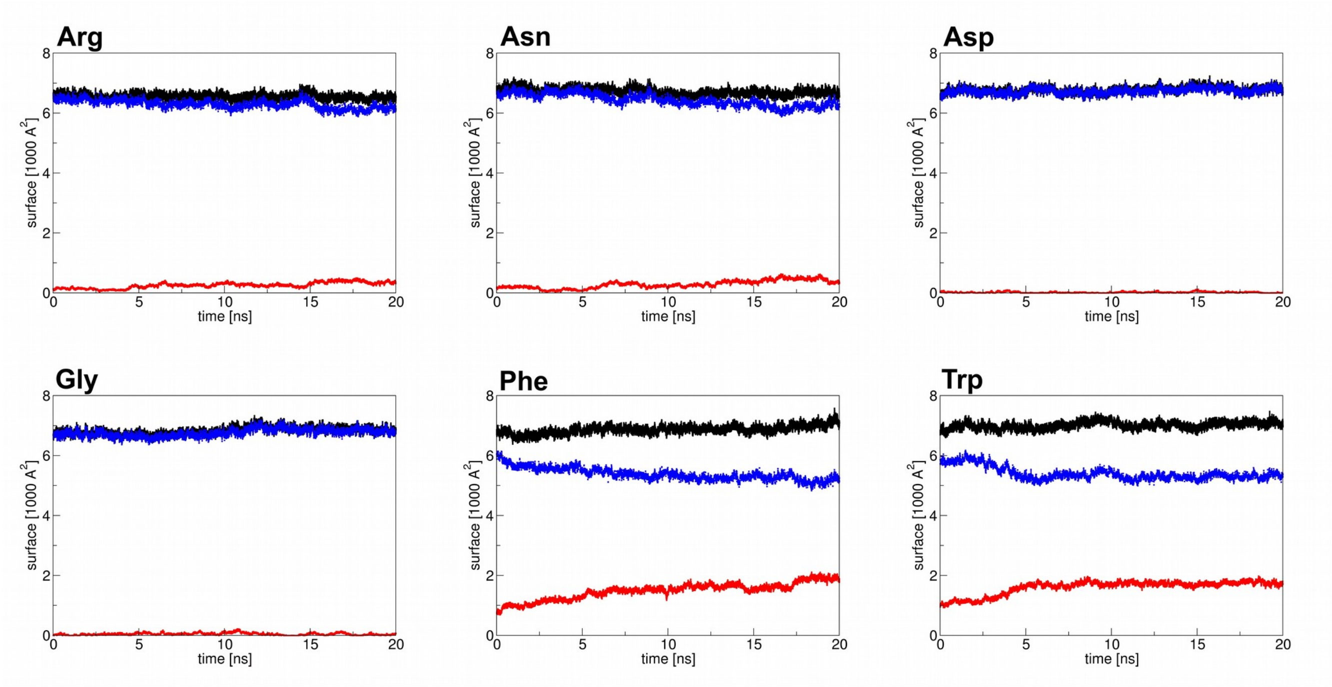

Figure S21. Time development of the total surface area (black), and surface coverages of the ***PET*** *nanoparticle by water (blue) and the amino acids (red) in the molecular dynamics simulation.*

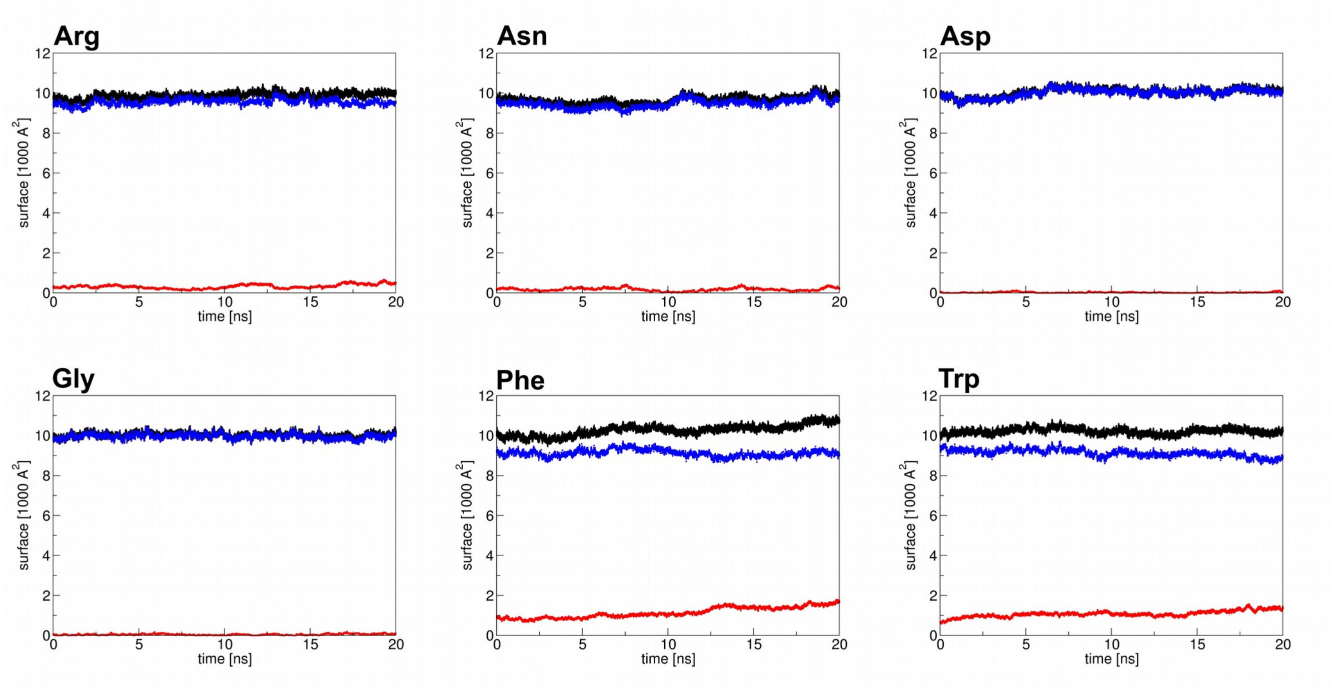

Figure S22. Time development of the total surface area (black), and surface coverages of the ***nylon-6,6*** *nanoparticle by water (blue) and the amino acids (red) in the molecular dynamics simulation.*
